# Perceptions of employees with a low and medium level of education towards workplace health promotion programmes: a mixed-methods study

**DOI:** 10.1186/s12889-022-13976-2

**Published:** 2022-08-25

**Authors:** Hanne C. S. Sponselee, Willemieke Kroeze, Suzan J. W. Robroek, Carry M. Renders, Ingrid H. M. Steenhuis

**Affiliations:** 1 Department of Health Sciences, Faculty of Sciences, VU University Amsterdam and Amsterdam Public Health Research Institute, 1081 HV Amsterdam, The Netherlands; 2 Care for Nutrition and Health Group, School of Nursing, Christian University of Applied Sciences, 6717 JS Ede, The Netherlands; 3 Department of Public Health, Erasmus University Medical Center, P.O. Box 2040, 3000 CA Rotterdam, The Netherlands

**Keywords:** Occupational health, Workplace health promotion programme, Prevention, Socioeconomic health inequities, Perceptions, Participation

## Abstract

**Background:**

Understanding the perceptions of lower socioeconomic groups towards workplace health promotion is important because they are underrepresented in workplace health promotion activities and generally engage in unhealthier lifestyle behaviour than high SEP groups. This study aims to explore interest in workplace health promotion programmes (WHPPs) among employees with a low and medium level of education regarding participation and desired programme characteristics (i.e. the employer’s role, the source, the channel, the involvement of the social environment and conditions of participation).

**Methods:**

A mixed-methods design was used, consisting of a questionnaire study (*n* = 475) and a sequential focus group study (*n* = 27) to enrich the questionnaire’s results. Multiple logistic regression analysis was performed to analyse the associations between subgroups (i.e. demographics, weight status) and interest in a WHPP. The focus group data were analysed deductively through thematic analysis, using MAXQDA 2018 for qualitative data analysis.

**Results:**

The questionnaire study showed that 36.8% of respondents were interested in an employer-provided WHPP, while 45.1% expressed no interest. Regarding subgroup differences, respondents with a low level of education were less likely to express interest in a WHPP than those with a medium level of education (*OR* = .54, 95%, *CI* = .35–.85). No significant differences were found concerning gender, age and weight status. The overall themes discussed in the focus groups were similar to the questionnaires (i.e. the employer’s role, the source, the channel, the involvement of the social environment and conditions of participation). The qualitative data showed that participants’ perceptions were often related to their jobs and working conditions.

**Conclusions:**

Employees with a medium level of education were more inclined to be interested in a WHPP than those with a low level of education. Focus groups suggested preferences varied depending on job type and related tasks. Recommendations are to allow WHPP design to adapt to this variation and facilitate flexible participation. Future research investigating employers’ perceptions of WHPPs is needed to enable a mutual understanding of an effective programme design, possibly contributing to sustainable WHPP implementation.

## Background

Overweight and obesity prevalence is increasing worldwide [1]. In high-income countries, people with a low socioeconomic position (SEP) generally have a lower life expectancy and live in good health for a shorter duration than high SEP groups, mainly as a result of unhealthier behaviour [2, 3]. One of the reasons for this socioeconomic health inequity is that overweight and obesity prevalence is significantly higher amongst individuals with a low SEP than those with a high SEP [4]. Health promotion programmes that focus on promoting healthy lifestyle behaviour can contribute to the prevention of overweight and obesity [5]. If these programmes are specifically designed for people with a low SEP who have the greatest health potential [6], this approach can contribute to reducing socioeconomic health inequities [7].

The workplace is an important setting for focusing on promoting healthy behaviours. Most of the global population participates in the labour force, which allows for using existing social connections and reaching large groups [8, 9]. A systematic review indicated that workplace health promotion activities could contribute to positive changes in weight-related outcomes of employees [10]. Improvement of weight status might have positive results for employees and employers as it might prevent absenteeism due to illness, overall impairment at work and early exit from paid employment [7, 11, 12].

Employees with a low SEP are commonly acknowledged to often exhibit disadvantageous health statuses compared to those with a high SEP [13] and are more likely to experience unfavourable working conditions [14]. For instance, employees with a low SEP are more likely to do shift work associated with an increased risk of overweight and obesity [15, 16]. Unhealthy lifestyle behaviours (i.e. physical inactivity, improper diet and poor sleep quality) have been proposed to mediate the relationship between shift work and obesity [17]. Employees with a low SEP primarily engage in daily physical activity at work [18]. Higher doses of occupational physical activity appear to be less healthy than lower doses [19]. A meta-analysis demonstrated a relationship between long working hours and the incidence of diabetes, exclusively amongst employees of lower SEP [20]. Additionally, employees with a low SEP are often underrepresented in workplace health promotion research [21], partially explained due to workplace health promotion programmes (WHPPs) being commonly provided to employees with a high SEP [22].

The evidence base for socioeconomic health inequities concerning participation in health promotion programmes is inconsistent. A recent meta-analysis found no socioeconomic inequities in programme compliance (i.e. programme adherence), although the authors specifically emphasised the need to improve WHPPs for employees with a lower SEP [23]. Similarly, another study failed to establish a consistently lower level of initial or sustained participation amongst employees with a low level of education [24]. Other studies have shown that initial participation and compliance in such programmes are generally lower amongst employees with a lower SEP [25, 26].

Besides SEP, other employee characteristics have been linked to WHPP participation. Specifically, research has found that women [27, 28] and older employees [24] are more likely to participate in WHPPs. Furthermore, employees with a healthy weight and overweight have a greater likelihood of participating in such programmes than employees with obesity [28].

Concerning effectiveness, a recent meta-analysis showed that WHPPs produced little to no effect on health behaviour [23], possibly the result of generally low levels of compliance, which underscores the importance of understanding WHPP participation. This finding suggests it is important to gain insight into their perceptions of WHPP participation and programme characteristics to understand employee characteristics that facilitate or hinder participation [29]. Prior studies have shown that participants of WHPPs should be involved in the design process to enable successful recruitment [30] and improve the programmes’ reach and compliance [31]. Tailoring an intervention’s message, source, and channel to its users has also been crucial in enhancing its effectiveness in obesity prevention and reduction amongst people with a low SEP [32, 33].

Ideally, workplace health promotion must focus on the working environment and the individual (i.e. employee) [34]. This study has a focus on the perceptions of employees towards participation in the lifestyle component of a WHPP (i.e. eating behaviour and physical activity), and is embedded in a larger research project which aims to promote blue-collar employees’ health through both exploring the working environment [35] and employees’ lifestyle behaviour.

The aim of this paper is two-fold. First, we aim to explore associations between characteristics of employees with a low and medium level of education and their level of interest in WHPP participation. The second aim is to explore the perceptions of employees with a low and medium level of education to gain an in-depth understanding of the underlying reasons.

## Methods

### Design

This paper describes a mixed-methods design comprising two studies. The goal was to combine two types of information on WHPP perceptions of employees with a low and medium level of education. Study 1 consisted of a questionnaire designed to identify these employees’ characteristics and perceptions of WHPPs. Subsequently, in study 2, focus group discussions were conducted with another sample to enrich the results highlighted in study 1.

The reason for collecting sequential quantitative and qualitative data was to first gain a general idea about these employees’ perceptions through a large quantitative sample, then explore and explain these results in detail by discussing them in focus groups. The methods and results of study 1 are first described, followed by those emerging from study 2. Then, a general discussion is presented.

### Study 1: Questionnaire study

### Design, respondents and procedures

This study had a cross-sectional design that aimed to 1) explore characteristics of employees with a low and medium level of education related to their level of interest in WHPP participation and 2) identify their perceptions regarding WHPP participation. The inclusion criteria were being 18 years or older, being employed, having sufficient command of the Dutch language to complete a questionnaire, and having a low or medium level of education (i.e. at the most, secondary vocational education level 4, representing middle-management and specialist training). After the initial questionnaire development, it was pilot tested amongst a small convenience sample (*n* = 4) of employees with a low level of education in a real-life work setting. They were recruited by a contact person at one of the workplaces and agreed to participate in the questionnaire study using a flyer provided by the researcher, HS. These four employees did not participate in the questionnaire study.

The researcher read the questionnaire aloud, and respondents verbalised their thoughts while answering the questions according to the ‘thinking aloud’ method [36] to verify the comprehensibility and readability. Consequently, the questionnaire was revised to a B1 language level by a Dutch linguistic company in consultation with the researchers to preserve the original items’ meaning and indicate face validity. In general, a B1 language level is understood by 95% of the people living in the Netherlands [37].

Subsequently, the questionnaire was completed by two groups of employees to reach a large number of respondents. The first group comprised an online sample (*N* = 255) drawn from a data collection agency (i.e. Flycatcher), which maintains an online panel of more than 10,000 Dutch members who voluntarily participate in online surveys. Panel members receive credits exchangeable for a gift voucher per completed questionnaire.

The second group was a face-to-face sample consisting of respondents with blue-collar jobs in eight organisations in the Netherlands (*N* = 220). The organisations covered a diverse range of sectors: healthcare (*n* = 3), construction (*n* = 1), public sector (*n* = 1), infrastructure (*n* = 1), civil engineering (*n* = 1), and logistics (*n* = 1). One international and seven national organisations were represented, situated in urban and regional areas. Respondents completed the questionnaire individually at the workplace, either in a private room or with other respondents. HS was present in both settings to answer questions.

### Measurements

#### Perceptions of workplace health promotion programmes

The question *‘Do you want your employer to offer you a workplace health promotion programme?’* was answered with a) No, absolutely not, b) No, preferably not, c) I do not know, d) Yes, that would be fine or e) Yes, certainly. The responses were then categorised into ‘No interest or not sure’ (a, b and c) and ‘Interested’ (d and e).

Questions regarding the respondents’ perceptions of WHPPs were developed based on insights from health-promoting interventions and health communication strategies (i.e. message, source, channel) [32, 33]. They were categorised into the themes: 1) preferences regarding the employer’s role in providing a WHPP, 2) the programme delivery source, 3) the channel of programme delivery, 4) participation of the social environment and 5) conditions for participation. Items were introduced via a short explanation of the proposed WHPP: *‘a programme in which you will learn about or engage in healthy eating, body weight and being physically active’.* Each theme contained a short introduction, for example, for the channel of programme delivery theme: *‘The following questions are about ways to receive a health promotion programme at work’.* Questions contained five similar answer options as described at the start of this paragraph (i.e. ranging from no to yes), aligned with the exact phrasing of the question.

#### Respondent characteristics: Demographics and body mass index

Demographic characteristics related to gender, age and the highest level of education were obtained. The level of education was used as a proxy for SEP and was classified into two levels per the Dutch standard classification of education [38]: low (at the most secondary vocational education level 1) and medium (at the most secondary vocational education level 4). Self-reported data on height (in centimetres, without shoes) and weight (in kilograms) were also gathered. The body mass index (BMI) score was calculated by dividing weight into kilograms by the square of the height in metres and divided into three weight status categories: healthy weight (18.5–25.0 kg/m^2^), overweight (25.0–30.0 kg/m^2^) and obese (≥ 30.0 kg/m^2^).

### Data analyses

Data were analysed using SPSS Statistics 27.0. Four outliers for BMI (BMI > 51.59 kg/m2) were excluded during data cleaning. Five BMI values below the range of the healthy weight category (i.e. < 18.5 kg/m^2^ [39], range 15.2–18.2) were excluded since grouping these five values did not qualify as a separate category. Two cases missing 41.2% and 55.9% of the values were also excluded.

Complete case analysis was carried out, and the total sample was described using descriptive analyses. Associations between demographics and weight status subgroups concerning interest in WHPPs were analysed utilising a multiple logistic regression, and its required assumptions were met. Finally, descriptive analyses were conducted to describe the perceptions of those interested in a WHPP.

## Results

A total of 475 respondents with a low and medium level of education were included in the analysis (i.e. 255 online sample members, 220 face-to-face sample members). The two samples differed based on demographic characteristics (i.e. gender, age, level of education). In total, 51.3% were women, and 48.7% were men. The mean age was 48.5 years (*SD* = 12.3).

Furthermore, 69.3% had a low level of education, while 30.7% had a medium level of education. The mean BMI was 26.7 kg/m^2^ (*SD* = 4.9), of which 59.8% belonged to the overweight or obese weight status subgroup. In total, 36.8% of the respondents were interested in a WHPP provided by their employer, while 45.1% expressed no interest (Table 1). Additionally, 18.9% reported they did not know whether they were interested.

**Table 1:** Characteristics of respondents regarding interest in a workplace health promotion programme provided by the employer

		N interested	N not interested	N I do not know	N total
	Subgroup				
**Gender**	Men	98 (42.4%)	87 (37.7%)	46 (19.9%)	231 (100%)
	Women	77 (31.7%)	127 (52.3%)	39 (16.0%)	243 (100%)
*N*		175	214	85	474
**Age**	< 40 years	46 (42.2%)	42 (38.5%)	21 (19.3%)	109 (100%)
	≥ 40 years	127 (35.3%)	169 (46.9%)	64 (17.8%)	360 (100%)
*N*		173	211	85	469
*M* ± *SD*		47.5 ± 12.3	49.6 ± 12.2	48.2 ± 12.5	48.5 ± 12.3
**Level of education**	Low	106 (32.2%)	167 (50.8%)	56 (17.0%)	329 (100%)
	Medium	69 (47.3%)	48 (32.9%)	29 (19.9%)	146 (100%)
*N*		175	215	85	475
**Weight status**	Healthy weight	56 (32.6%)	90 (52.3%)	26 (15.1%)	172 (100%)
	Overweight	77 (40.5%)	82 (43.2%)	31 (16.3%)	190 (100%)
	Obese	35 (37.2%)	37 (39.4%)	22 (23.4%)	94 (100%)
*N*		168	209	79	456
*M* ± *SD* (kg/m^2^)		27.1 ± 4.7	26.3 ± 4.9	27.4 ± 4.8	26.7 ± 4.9

Table 2 shows that the likelihood of being interested in a WHPP was lower amongst respondents with a low level of education than those with a medium level of education (*OR* = 0.54, 95% *CI* = 0.35–0.85). No significant differences were found concerning gender, age or weight status groups.

**Table 2:** Characteristics of respondents with an interest in a WHPP (N = 175), by multiple logistic regression analysis

Interest in participating in a workplace health promotion program – ‘Interested’ versus ‘No interest or not sure’		
	**OR**	**95% CI**
**Variables **^**1**^		
Gender: Men	1.46	.97–2.19
Age: < 40 years	1.06	.64–1.76
Level of education: Low	.54^*^	.35-.85
Weight status: Overweight	1.47	.94–2.30
Weight status: Obese	1.32	.76–2.29

^*^p < 0.05

^1^ Reference groups: gender = women; age =  ≥ 40 years; level of education = medium; weight status = healthy weight

Respondents’ perceptions who expressed an interest in WHPP participation (i.e. the ‘Interested’ group) appear in Table 3. The category *preferences regarding the employer’s role in providing a WHPP* shows that most respondents were interested in their employer providing a cooking course or a healthcare specialist supporting or facilitating healthy lifestyle behaviour (ranging from 63.4% to 77.1%; Table 3).

**Table 3:** Perceptions regarding a workplace health promotion program among those in the ‘Interested’ group (*N* = 175)

	*Yes N(%)*	*No N(%)*	*I do not know N(%)*
**Preferences regarding the role of the employer in providing a WHPP**			
Providing a cooking course	135 (77.1%)	25 (14.3%)	15 (8.6%)
Providing support by a health care specialist	131 (74.9%)	28 (16.0%)	16 (9.1%)
Facilitating healthy eating at work	130 (74.7%)	16 (9.2%)	28 (16.1%)
Facilitating weight management	111 (63.8%)	28 (16.1%)	35 (20.1%)
Facilitating physical activity	111 (63.4%)	31 (17.7%)	33 (18.9%)
**Program delivery source**			
Health care specialist such as a dietician, physiotherapist or lifestyle coach	134 (77.0%)	29 (16.7%)	11 (6.3%)
Confidential counsellor	89 (51.1%)	49 (28.2%)	36 (20.7%)
Colleagues who have a healthy lifestyle	71 (40.8%)	60 (34.5%)	43 (24.7%)
**Channel for program delivery**			
Individual counseling with a coach at work	98 (57.3%)	52 (30.4%)	21 (12.3%)
Course with colleagues and a coach at work	83 (48.5%)	62 (36.3%)	26 (15.2%)
Individual counseling, web-based	70 (40.0%)	73 (41.7%)	32 (18.3%)
Course with colleagues, web-based	42 (24.4%)	99 (57.6%)	31 (18.0%)
**Participation of social environment**			
Involving a colleague	82 (51.9%)	32 (20.3%)	44 (27.8%)
Involving their partner	67 (48.2%)	31 (22.3%)	41 (29.5%)
Involving a friend	58 (38.2%)	47 (30.9%)	47 (30.9%)
Involving a family member	50 (33.6%)	51 (34.2%)	48 (32.2%)
	*(Very) important, N(%)*	*A little bit important, N (%)*	*(Totally) not important, N(%)*
**Conditions for participation**			
Employer pays for the program	117 (66.9%)	37 (21.1%)	21 (12.0%)
Employer is not able to access personal information	97 (55.7%)	39 (22.4%)	38 (21.8%)
Following the programme during working hours	93 (53.4%)	44 (25.3%)	37 (21.3%)

Concerning the *programme delivery source*, a healthcare specialist (77.0%) and a confidential counsellor (51.1%) scored highest. Concerning the *channel of programme delivery*, most respondents answered positively about receiving the WHPP through individual counselling with a coach at work (57.3%). Almost half the respondents (48.5%) were positive about receiving the WHPP in a course with colleagues and a coach at work.

In contrast, fewer respondents responded positively to a web-based WHPP involving one-to-one counselling (40.0%) or colleagues (24.4%). Concerning the *participation of the social environment,* between 33.6% (a family member) and 51.9% (a colleague) of the respondents answered that they would want to involve their social environment in the WHPP. Regarding *conditions for participation,* most respondents answered that it was either very important or important that their employer paid for the WHPP (66.9%). More than half the respondents (55.7%) answered that it was very important or important that the employer could not access any of their personal information, while the remaining half considered it to be of little or no importance. Similarly, more than half the respondents (53.4%) answered that it was either very important or important that they could attend the programme during working hours, while the other half considered it to either be of little or no importance.

### Study 2: Focus group study

### Design, study population and recruitment procedures

This study utilised a qualitative design using focus groups to understand why employees with a low and medium level of education had certain perceptions towards WHPPs. The inclusion criteria were being 18 years or older, having sufficient command of the Dutch language to participate in a group discussion, and having a blue-collar job. The criterion of having a blue-collar job was used instead of having a low or medium level of education because the two are typically associated and to avoid stigmatisation. To reach these employees, we first identified industries with a substantial number of blue-collar employees (e.g. construction, healthcare, hospitality industries) using a Dutch report on the labour market position [40]. A list of organisations within these industries in the Netherlands was made through purposive sampling. The organisations’ human resource managers or service desks were contacted by email and telephone.

Four national medium-to-large organisations in urban areas agreed to facilitate employee recruitment. They appointed contact persons unknown to the research team prior to the study, with managerial positions related to the employees. These contact persons approached and informed blue-collar employees of the request to voluntarily participate by distributing flyers in designated announcement spots such as information boards. Flyers explicitly emphasised the voluntary nature of study participation and described the incentive of a €15 gift voucher (provided by HS). Five focus groups were held: two in one public sector organisation (i.e. a property management team, *n* = 4 and an audiovisual team, *n* = 7), one in a service sector organisation (i.e. a catering team, *n* = 6) and two focus groups in two healthcare organisations (i.e. domestic workers team, *n* = 6 and a care assistant team, *n* = 4).

### Data collection and procedures

An interview guide was developed based on the main themes emerging from the questionnaire in study 1 (i.e. preferences regarding the employer’s role, the programme delivery source, the channel of programme delivery, the participation of the social environment and conditions for participation). The discussions started with, ‘How would you like to participate in a lifestyle programme at work?’ This question was supplemented by open-ended follow-up questions about why, how and under which conditions the participants would envisage participating in a WHPP, based on the main results of study 1. The four authors formulated these follow-up questions after familiarising themselves with the main results of study 1 and reached a consensus on topics that required further exploration.

The focus groups were organised between May and July 2019 at the participants’ workplaces for practical reasons (e.g. a low threshold for participation during working hours for participants and employers). Discussions took place in private rooms without employers to ensure participants could speak freely, following focus group guidelines [41]. The discussions lasted between 57 and 69 min and were recorded (Olympus WS-853).

First, HS emphasised confidentiality and explained the study background, including WHPP examples supported by visual aids. Then, all participants gave written informed consent. Visual aids representing the main topics were visible to all participants during the discussions. A research assistant observed the atmosphere and took notes. After the discussions, the participants completed a questionnaire on demographics (i.e. gender, age, and the highest education level) and weight status (i.e. height and weight).

### Data analyses

The discussions were transcribed verbatim by a research assistant and subsequently verified by HS, randomly comparing pieces of audio and text for each transcript. First, two researchers familiarised themselves with the research assistant’s notes and transcripts. Then, they analysed the transcripts using the MAXQDA 2018 qualitative data analysis software package. Data were analysed deductively through thematic analysis [42] while using a broad framework for the coding process [43, 44]. The two researchers independently carried out the thematic analysis by deductively generating initial codes within the main categories. The categories were similar to the interview guide themes.

Next, they discussed any differences in a consensus meeting, resulting in a coding tree. Finally, HS and IS discussed and agreed on the final codes. For example, the theme ‘conditions for participation’ contained the main code ‘costs’, including the subcodes ‘employer should pay’ and ‘payment by employer not important’. HS and IS agreed on reaching data saturation since they considered the data provided adequate insight, and no new themes emerged in the last focus group [40].

## Results

A total of 27 participants with blue-collar jobs were involved in the five focus groups: 13 were women, and 14 were men. The mean age was 46.8 years, ranging from 27 to 59 years (*SD* = 8.4). Regarding the level of education, 13 participants had a low level, 13 had a medium level, and one had a high level of education. Participants had a mean BMI of 23.3 kg/m^2^, ranging from 18.1 to 36.1 kg/m^2^ (*SD* = 4.6). Table 4 presents the overall results.

**Table 4:** Perceptions regarding a workplace health promotion program: focus group resul﻿ts

Category	Topics mentioned
**Preferences regarding the role of the employer in providing a WHPP**	**General attitude regarding the role of the employer** • Lifestyle behaviour is own responsibility — Woman, catering team member, service sector organization (participant 2): ‘*Yes, you know, you have to know yourself how your style of life is. If you want to go to the pub every night and drink beers, that's your choice. That's your own responsibility’* • Own motivation is key to change lifestyle • Preference to keep work and private life separate — Woman, domestic worker, healthcare organization (participant 6): ‘I just *prefer to arrange things myself. It doesn't have to be through work. I am like: I do my work and for the rest I take care of my things’* **Negative attitude towards the employer providing the program** • When employer provides this, it feels like an obligation to participate — Woman, domestic worker, healthcare organization (participant 1): *‘If it is offered and you don't participate and a few programmes are provided and every time you don't participate because it, then you get an uncomfortable feeling like, soon they will think: you don't participate anywhere. […] What I'm afraid of is, if you do go along with it, it's not compulsory, but they do offer it and you get sick that you'll be told: 'Yes, but you're not participating either'* **Positive attitude towards the employer providing the program** • Employer is allowed to provide it
**Program delivery source**	• The employer
	• Health care specialist or lifestyle coach **Reason for a negative attitude towards a health care specialist** - Already seeing a health care specialist such as a physiotherapist or dietitian **Reasons for a positive attitude in favour of a health care specialist** - As support - To gain knowledge - Because of gaining weight - Getting support for improving physical activity
	• A colleague outside the team
**Channel for program delivery**	The following channels were mentioned by the participants: • Web-based, because it is user-friendly and commonly used. Suggestions: - Mobile app - E-learning - E-mails - Combining web-based channels, such as E-learning with e-mails Woman, care assistant, healthcare organization (participant 4)*: ‘You can also do both. I mean using the e-learning once in a while. And then you can send some information now and then which you read again and then you're like: 'Oh yes.'*
**Participation of social environment**	Colleagues, partner, family, neighbours, and friends were mentioned regarding a person from their social environment whom could participate in the program. Furthermore, the results revealed that: • Participation of a person from the social environment is motivating, especially in physical activity • It is up to the persons (i.e. family members, neighbours, friends) themselves to decide. They should be motivated — Woman, catering team member, service sector organization (participant 1): *‘It's hard to think for people anyway’* — Man, property management team member, public sector organization (participant 2). *‘That person has to decide that for themselves, I have to think about myself’*
**Conditions for participation**	Participants perceived the condition of costs in the following way: • Some participants mentioned the employer should pay • Others mentioned it is not important whether the employer pays for it
	Participants perceived the condition of privacy in the following way: • Some participants mentioned the employer is allowed to know everything • Others mentioned colleagues should not see each other’s personal progress
	Participants perceived the condition of time of day dependent on their type of job and related tasks: • Some participants suggested participation directly after working hours • Others mentioned preferring participation during working hours — Man, property management team member, public sector (participant 1): *‘If it's during working hours then you think: yeah, well, a little bit of relaxation […]. Then I still like it, you know. But if you have to do it after working hours, well, then you go, you arrive at a certain time, you go home for dinner first and then you have to be able to get up and say: OK, I'm going to go out and do some sports. And we are getting to a certain age where it is more and more difficult to do that.’* — Man, property management team member, public sector (participant 3): *‘For me it is not like that. I think it is too much hassle. To do it during working hours’*

### Preferences regarding employer’s role in providing a WHPP

Participants mentioned various factors related to their employer’s role in providing a WHPP. The workload was mentioned because many participants found it challenging to imagine participating in such a WHPP due to their job demands. According to a male audio-visual team member at a public sector organisation (participant 2):

Well, I am trying to visualise how that could exist in our dynamic work area, so to speak. We sometimes have to go in and out, and in between, you plan your lunch break, whether you want to or not.

Furthermore, some participants reported that their physically demanding jobs affected their private lives to the extent that they did not have the time to participate in a WHPP outside of working hours. Many participants also indicated that their lifestyle behaviour was their responsibility instead of their employer’s. They emphasised that it is a personal choice to live a healthy life, which is different for everyone. They often added that their motivation was the key component to lifestyle change, and they wanted to keep their work and private lives separate. They also noted that they would feel obliged to participate if their employer provided a WHPP. In contrast, others stated they would be fine if their employer provided a WHPP, either because it would be fun or because they already lived a healthy lifestyle. Thus, participants mentioned factors that appeared to be interrelated (i.e. job type, lifestyle and responsibility).

### Programme delivery source

Several WHPP programme delivery sources were referenced. Some participants noted that a colleague outside the team, solely involved in supporting employees’ lifestyles, would be helpful. Additionally, a lifestyle coach was mentioned as a useful provider to give professional advice. According to a female domestic worker in a healthcare organisation (participant 5):

In the sense of, say, a coach who knows you, if you have a bad lifestyle, that they can say, ‘OK, well, I’ve got a piece of advice for you. Would you maybe do it like this and like that?’

Based on the results of study 1, the researcher posed the option of either an employer or a healthcare specialist providing the programme. Support from a healthcare specialist was important to some. Reasons were gaining nutritional knowledge, living healthier because they were currently gaining weight, and improving physical activity. Participants who expressed no interest in receiving support from a healthcare specialist mentioned that they were currently receiving or had previously received counselling from a dietician or physiotherapist. Thus, preferred programme delivery sources varied, although the primary reason was receiving support.

### Channel of programme delivery

Participants were asked how they preferred to receive a WHPP. Web-based channels were the most frequently mentioned, including mobile applications, e-learning and emails. The reasons for preferring web-based delivery channels were that they were the most user-friendly method and that online tools are commonly used nowadays. The preference for a specific delivery channel depended on the channel the participants were used to working with. According to a male care assistant in a healthcare organisation (participant 1)*:*

*Well, we are already used to working with e-learning in the industry or the digital platform on which you learn something about each subject. So, that would be the easiest thing for everyone, because then you just say, ‘This is the lifestyle part, so do this part’.*

Some participants preferred a combination of web-based channels, such as e-learning and emails. One participant preferred to use a hardcover book to gain easier access to information. Participants who expressed a willingness to participate in the physical activity component of a WHPP mostly preferred doing so at work or a gym. Participants generally favoured an online programme, except for the physical activity component.

### Participation of the social environment

Participants were asked whether they preferred to involve a person from their social environment in the WHPP. Participants stressed that they could not think for them and that those persons should decide for themselves if they wished to participate. Some participants said they could not think of a preferred person from their social environment, while others who expressed a preference mentioned their partner, family, neighbours or friends. Participants did not specifically indicate whose participation they preferred but said that their participation would motivate them to engage in the physical activity component of a WHPP.

The researcher added that perhaps colleagues could also participate. Some participants responded that it would be fun, while others said it would be impossible due to employees’ broad range of job tasks. The following quote concerns how a person from the participant’s social environment could be involved, according to a male care assistant in a healthcare organisation (participant 3):

*Well, you just have to talk to them about it like, ‘Well, I’ve got something going on at work and this and that’. And explain a bit, maybe if you notice that they are interested in it you can say: ‘Hey, isn’t it something for you to participate in? Or that you can support me in something’.*

Overall, no common idea regarding this involvement existed, and the willingness of the other person to participate in the programme was deemed crucial.

### Conditions for participation

Some conditions discussed were costs, privacy and time of day. While some participants stated it was important that their employer paid for the programme, others did not perceive it as essential. Regarding privacy, several participants reported that they would agree with their employer knowing everything about them, while others considered privacy crucial because they did not want their colleagues to see their programme progress.

Concerning the time of day, some participants expressed interest in attending immediately after working hours, while others said the programme should be provided during working hours. This preference depended on the type of job and the tasks involved. Overall, this theme illustrated that their perceptions varied widely for each condition.

## Discussion

This paper firstly aimed to explore associations between characteristics of employees with a low and medium level of education and interest in WHPP participation. The second aim was to explore their perceptions to gain an in-depth understanding of the underlying reasons.

### Characteristics of employees who expressed an interest in participating in WHPPs

Regarding employee characteristics, we found that employees with a medium level of education were more likely to be interested in participating in a WHPP than those with a low level. One explanation was that employees with a low level of education were more likely to work in jobs with disadvantageous working conditions, such as shift work and high physical activity [14, 18], which might have been a barrier to being interested in WHPP participation. The focus group results supported the impact of unfavourable working conditions on employee perceptions. Another explanation was that we explored employees’ hypothetical interest in participation, which might have been more abstract to practice-oriented employees with a low level of education than those with a medium level.

Although previous studies have found associations between being a woman [27, 28], being older [24], having a healthy weight or overweight weight status [28] and WHPP participation, our study did not find these associations concerning interest in WHPP participation. These aforementioned studies included people with a high SEP. Therefore, their results might not be comparable to our findings. Furthermore, we did not study actual participation but focused on the intention to participate under certain proposed conditions. Intention has frequently been described as a proxy for actual behaviour [45, 46], though a relationship between intention and WHPP participation has not always reflected actual participation [27, 47].

### Perceptions regarding WHPPs

Overall, over one-third of respondents in study 1 expressed an interest in participating in an employer-provided WHPP. This finding was comparable to the median participation of 33% found in a review on initial participation in a WHPP, where participation levels varied from 10 to 64%, and people with a high level of education were included [48]. In our questionnaire study almost half the respondents showed no interest in participating in a WHPP. Moreover, the results from the focus groups indicated that their limited interest was related to a high workload.

Limited interest in WHPP participation may partly be explained by job demands and the working environment. First, physically demanding work is an inherent feature of many blue-collar jobs [18], typically performed by employees with a low or medium level of education. These high physical demands might have influenced the level of interest in WHPPs with a physical activity component. Second, the limited interest in WHPP participation might have been influenced by a broad range of working environment factors. Organizational culture can hinder participation if it does not prioritize health promotion [49, 50]. Furthermore, the physical working environment plays an important role, as the presence of health-promoting facilities (e.g., a kitchen where employees can prepare food) can contribute to healthier behaviour [49].

Our results also indicated that the participants were positive about a healthcare specialist as the WHPP source or provider. Commonly mentioned reasons were the possibility of receiving support and gaining knowledge from this specialist. Similarly, a study in an Australian transport company found that personal communication and the physical presence of a healthcare specialist (i.e. a registered dietitian or exercise physiologist) were evaluated highly by the employees participating in such programmes [29].

Concerning the preferred channel of programme delivery, no consensus emerged. For instance, receiving the WHPP at work scored higher in the questionnaire study than web-based options, but web-based channels were more frequently mentioned in the focus groups because they were perceived as user-friendly and commonly used. Prior research might help to explain these divergent findings, as online channels have previously been viewed as incredibly convenient [51], while face-to-face options have been shown to enable group interaction components [52]. Another explanation was that the perceptions regarding preferred channels might have been related to possible working environments.

Regarding participation from their participant’s social environment, no clear preference was found in the questionnaire study as to who this person should be. The focus group study might partially explain this finding. Participants considered that including someone from their social circle would motivate them to participate, without specifying whose participation they preferred. This finding was confirmed by a study that stressed the importance of social support to stimulate behaviour change and increase self-efficacy [53].

However, participants also argued that the decision to have someone from their social environment participate was not theirs but the person’s. From this perspective, one could argue that answering this question might have been challenging. This difficulty might explain why more than a quarter of respondents in the questionnaire study answered ‘I do not know’ to this theme.

The last theme involved conditions for participation. In the questionnaire study, most respondents reported it was either important or very important that their employer paid for the WHPP. Previous research has demonstrated that cost concerns should be considered when delivering lifestyle interventions to adults with a low SEP [54]. The focus groups showed a greater variety of opinions, perhaps because they frequently wished to keep work and their private lives separate. This outcome might have been related to the desire to pay for the programme themselves.

Moreover, preferences regarding the time of day varied throughout the questionnaire and focus group studies, which might be explained by the focus group findings, in which a preference for the time of day seemed to be highly dependent on the type of job and related tasks.

### Strengths and limitations

To the best of our knowledge, this is the first study of its kind to use a mixed-methods design to explore the characteristics of employees with a low and medium level of education related to interest in WHPP participation and their in-depth perceptions of WHPPs. This research design allowed for the triangulation of quantitative and qualitative data, which proved beneficial as the following focus groups revealed valuable underlying information. Therefore, the findings contribute to the bigger picture of understanding this population’s interrelated perceptions of work, health and lifestyle.

However, we asked employees to think hypothetically about whether they would be interested in participating in a WHPP. This request required abstract thinking, which occasionally might have been challenging for these practice-oriented employees. Nevertheless, we believe the results are valuable because we have tried to concretise them in the best possible way through additional descriptions in the questionnaire and visual aids during the focus groups. Some additional limitations are noted for both studies separately.

### Study 1: Questionnaire study

The questionnaire’s convergent, divergent, and criterion validity could not be tested because we did not compare it with another questionnaire or theoretical construct. However, the questionnaire’s face validity was shown, and its B1 language level was considered appropriate for the respondents because it is comprehensible to 95% of people living in the Netherlands [37]. Although the two samples of the questionnaire study differed based on demographic characteristics, this difference was justified by mutually reporting the results of both samples.

### Study 2: Focus group study

The qualitative results should be interpreted with caution as other settings (e.g. solely urban or regional areas) might achieve different results. The extent to which data saturation was obtained was difficult to justify as new themes might have emerged with participants at other organisations in different areas in the Netherlands. However, the findings suggested satisfactory data saturation because the fifth focus group did not reveal new themes.

### Recommendations

We found variability in employees’ perceptions, underscoring the need to incorporate sufficient space for flexible WHPP design to meet employees’ needs. Our study clarified that interest in WHPP participation might be more feasible for some employees when both the time and place are aligned with their type of job and working conditions. For example, some employees might benefit from partially participating during and after working hours, benefiting from their employer’s WHPP facilitation. Other studies have also recommended tailoring these programmes to employees’ needs [55] and working conditions [56, 57]. In addition, the physical working environment should be considered when designing WHPPs. For example, using nudging strategies in worksite cafeterias may guide employees’ purchasing behaviour toward healthier food choices [58].

Another possibility is participation at another location than the workplace. Although most occupational health initiatives currently target healthy functioning in the workplace [59], this point corresponds with a recent meta-analysis that recommended new directions for such health promotion activities [23]. Similarly, WHPP recruitment should match these blue-collar employees’ job and working conditions, as some teams might read recruitment flyers on the intranet while others might only find such flyers on notice boards in their workplace. Thus, we recommend that WHPP designers incorporate a flexible programme design and recruitment strategies to meet employees’ needs and working conditions.

Another recommendation concerns support from someone in the participant’s social environment and a healthcare specialist. Our results show that these types of support could be motivating factors for potential WHPP participation. Other studies have shown that employees with a high level of social support are more likely to have positive intentions toward WHPP participation [27], and individual coaching by an expert can be a significant weight loss predictor [60].

For example, a healthcare specialist could support employees by making consulting appointments based on their needs and schedules, which might suit their different needs regarding when they would like to participate in a WHPP. Furthermore, a healthcare specialist could focus on increasing the participants’ self-efficacy, which has been associated with a positive intention toward WHPP participation [27]. Tailored support might lower the threshold for WHPP participation and should be considered part of WHPP implementation.

Future research should explore the underlying reasons for educational differences regarding interest in WHPP participation in further depth. Exploring this group’s capabilities and possibilities in conjunction with their preferred programme characteristics might also yield a broader understanding of their perceived worlds. These insights could provide WHPP designers and implementers guidance on the appropriate WHPP design, recruitment and implementation that suits employees with different levels of education and working conditions. Subsequently, the possibility of contributing to reducing socioeconomic health inequities might expand.

Another area for future research might be exploring employers’ perceptions of sustainable WHPP implementation, associated implementation tools and delivery modes. Together with this study’s insights, these perceptions could lead to a mutual understanding of WHPPs according to employees and employers. This two-way comprehension could contribute to the effective design, development and sustainable implementation of such programmes.

## Conclusions

We observed that employees with a medium level of education were more inclined to be interested in WHPP participation than employees with a low level in the questionnaire study. No differences were found between gender, age and weight status concerning interest in a WHPP. Over one-third of the respondents expressed interest in a WHPP, while almost half displayed no interest. Many participants in the focus group study explained that their limited interest arose from having a demanding job and perceiving their lifestyle as their responsibility.

The studies together showed that a healthcare specialist was the preferred provider of the programme because of their ability to give support and knowledge. There was no commonly shared opinion regarding the preferred channel of programme delivery (i.e. web-based or offline). The stated preferences regarding WHPP conditions appeared to vary depending on the participants’ job types and job-related tasks. Therefore, a flexible WHPP design that allows for adaptation to various jobs and working conditions is recommended. Finally, future research could focus on employers’ perceptions of WHPPs to foster a mutual understanding of WHPP implementation. This focus could contribute to the effective design, development and sustainable implementation of such programmes.

## Data Availability

The datasets generated and analysed during the current study are not publicly available because study participants did not explicitly agree that their raw data would be shared publicly, but are available from the corresponding author on reasonable request.

## Abbreviations

BMI: Body mass index
SEP: Socioeconomic position
WHPP: Workplace health promotion programme

## References

[1] Abarca-Gómez L, Abdeen ZA, Hamid ZA, Abu-Rmeileh NM, Acosta-Cazares B, Acuin C (2017). Worldwide trends in body-mass index, underweight, overweight, and obesity from 1975 to 2016: a pooled analysis of 2416 population-based measurement studies in 128·9 million children, adolescents, and adults. The Lancet.

[2] Beckfield J, Olafsdottir S, Bakhtiari E (2013). Health Inequalities in Global Context. Am Behav Sci.

[3] Oude Groeniger J, Kamphuis CBM, Mackenbach JP, Beenackers MA, van Lenthe FJ (2019). Are socio-economic inequalities in diet and physical activity a matter of social distinction? A cross-sectional study. Int J Public Health.

[4] Marmot M (2005). Social determinants of health inequalities. The Lancet.

[5] Shaw K, O'Rourke P, Del Mar C, Kenardy J (2005). Psychological interventions for overweight or obesity. Cochrane Database Syst Rev.

[6] van Heijster H, Boot CRL, Robroek SJW, Oude Hengel K, van Berkel J, de Vet E (2021). The effectiveness of workplace health promotion programs on self-perceived health of employees with a low socioeconomic position: An individual participant data meta-analysis. SSM Popul Health.

[7] Burdorf A, Robroek SJW, Schuring M, Brouwer S, van Holland BJ, Koolhaas W, et al. Kennissynthese Werk(en) is Gezond: Een studie in opdracht van ZonMW. Rotterdam; 2016.

[8] World Bank. Labor force participation rate: International Labour Organization, ILOSTAT database; 2021 [Access date: 7 September 2021]. Available from: https://data.worldbank.org/indicator/SL.TLF.CACT.NE.ZS?end=2020&start=1990&view=chart].

[9] Holt-Lunstad J (2018). Fostering Social Connection in the Workplace. Am J Health Promot.

[10] Proper KI, van Oostrom SH (2019). The effectiveness of workplace health promotion interventions on physical and mental health outcomes - a systematic review of reviews. Scand J Work Environ Health.

[11] Shrestha N, Pedisic Z, Neil-Sztramko S, Kukkonen-Harjula KT, Hermans V (2016). The Impact of Obesity in the Workplace: a Review of Contributing Factors, Consequences and Potential Solutions. Curr Obes Rep.

[12] Robroek SJ, Reeuwijk KG, Hillier FC, Bambra CL, van Rijn RM, Burdorf A (2013). The contribution of overweight, obesity, and lack of physical activity to exit from paid employment: a meta-analysis. Scand J Work Environ Health.

[13] Robroek SJW, Rongen A, Arts CH, Otten FWH, Burdorf A, Schuring M (2015). Educational Inequalities in Exit from Paid Employment among Dutch Workers: The Influence of Health, Lifestyle and Work. PLoS ONE.

[14] Dieker AC, IJzelenberg W, Proper KI, Burdorf A, Ket JC, van der Beek AJ, et al. The contribution of work and lifestyle factors to socioeconomic inequalities in self-rated health ‒ a systematic review. Scand J Work Environ Health. 2019(2):114–25. 10.5271/sjweh.3772 10.5271/sjweh.3772 30370911

[15] Grundy A, Cotterchio M, Kirsh VA, Nadalin V, Lightfoot N, Kreiger N (2017). Rotating shift work associated with obesity in men from northeastern Ontario. Health Promot Chronic. Dis Prev Can.

[16] Liu Q, Shi J, Duan P, Liu B, Li T, Wang C (2018). Is shift work associated with a higher risk of overweight or obesity? A systematic review of observational studies with meta-analysis. Int J Epidemiol.

[17] Hulsegge G, Proper KI, Loef B, Paagman H, Anema JR, van Mechelen W (2021). The mediating role of lifestyle in the relationship between shift work, obesity and diabetes. Int Arch Occup Environ Health.

[18] Beenackers MA, Kamphuis CB, Giskes K, Brug J, Kunst AE, Burdorf A (2012). Socioeconomic inequalities in occupational, leisure-time, and transport related physical activity among European adults: a systematic review. Int J Behav Nutr Phys Act.

[19] Coenen P, Huysmans MA, Holtermann A, Krause N, van Mechelen W, Straker LM (2018). Do highly physically active workers die early? A systematic review with meta-analysis of data from 193 696 participants. Br J Sports Med.

[20] Kivimäki M, Virtanen M, Kawachi I, Nyberg ST, Alfredsson L, Batty GD (2015). Long working hours, socioeconomic status, and the risk of incident type 2 diabetes: a meta-analysis of published and unpublished data from 222 120 individuals. Lancet Diabetes Endocrinol.

[21] Stiehl E, Shivaprakash N, Thatcher E, Ornelas IJ, Kneipp S, Baron SL (2017). Worksite Health Promotion for Low-Wage Workers: A Scoping Literature Review. Am J Health Promot.

[22] Robroek SJW, Oude Hengel KM, van der Beek AJ, Boot CRL, van Lenthe FJ, Burdorf A (2020). Socio-economic inequalities in the effectiveness of workplace health promotion programmes on body mass index: An individual participant data meta-analysis. Obes Rev.

[23] Coenen P, Robroek SJW, van der Beek AJ, Boot CRL, van Lenthe FJ, Burdorf A (2020). Socioeconomic inequalities in effectiveness of and compliance to workplace health promotion programs: an individual participant data (IPD) meta-analysis. Int J Behav Nutr Phys Act.

[24] Robroek SJW, Lindeboom DEM, Burdorf A (2012). Initial and Sustained Participation in an Internet-delivered Long-term Worksite Health Promotion Program on Physical Activity and Nutrition. J Med Internet Res.

[25] Magnée T, Burdorf A, Brug J, Kremers SP, Oenema A, van Assema P (2013). Equity-specific effects of 26 Dutch obesity-related lifestyle interventions. Am J Prev Med.

[26] Craike M, Wiesner G, Hilland TA, Bengoechea EG (2018). Interventions to improve physical activity among socioeconomically disadvantaged groups: an umbrella review. Int J Behav Nutr Phys Act.

[27] Rongen A, Robroek SJ, van Ginkel W, Lindeboom D, Altink B, Burdorf A (2014). Barriers and facilitators for participation in health promotion programs among employees: a six-month follow-up study. BMC Public Health.

[28] Hall J, Kelly K, Burmeister L, Merchant J. Workforce Characteristics and Attitudes Regarding Participation in Worksite Wellness Programs. American journal of health promotion 2016;31. 10.4278/ajhp.140613-QUAN-283 10.4278/ajhp.140613-QUAN-283 26730552

[29] Street TD, Lacey SJ. Employee Perceptions of Workplace Health Promotion Programs: Comparison of a Tailored, Semi-Tailored, and Standardized Approach. Int J Environ Res Public Health. 2018;15(5). 10.3390/ijerph15050881 10.3390/ijerph15050881 PMC5981920 29710785

[30] Stuber JM, Middel CNH, Mackenbach JD, Beulens JWJ, Lakerveld J (2020). Successfully Recruiting Adults with a Low Socioeconomic Position into Community-Based Lifestyle Programs: A Qualitative Study on Expert Opinions. Int J Environ Res Public Health.

[31] van de Ven D, Robroek SJW, Burdorf A (2020). Are workplace health promotion programmes effective for all socioeconomic groups?. A systematic review Occup Environ Med.

[32] Kreuter MW, Wray RJ (2003). Tailored and targeted health communication: strategies for enhancing information relevance. Am J Health Behav.

[33] Coupe N, Cotterill S, Peters S (2018). Tailoring lifestyle interventions to low socio-economic populations: a qualitative study. BMC Public Health.

[34] European Network for Workplace Health Promotion. The Luxembourg Declaration on Workplace Health Promotion in the European Union. Luxembourg; 2018.

[35] Sponselee HCS, ter Beek L, Renders CM, Robroek SJW, Steenhuis IHM, Kroeze W. Exploring the implementation of individual and environmental health interventions in blue-collar work settings: stakeholders’ perceptions. In preparation. 10.3390/ijerph192013545 PMC9603088 36294131

[36] Ericsson KA, Simon HA (1980). Verbal reports as data. Psychol Rev.

[37] Bureau Taal. Bureau Taal. Eenvoudig Nederlands. [Access date: 5 March 2021]. Available from: http://www.bureautaal.nl/eenvoudig-nederlands-26].

[38] CBS. Standaard onderwijsindeling 2016. Den Haag/Heerlen: Centraal Bureau voor de Statistiek; 2017.

[39] World Health Organization. Obesity: preventing and managing the global epidemic. Report of a WHO consultation. 2000. Report No.: 0512–3054. 11234459

[40] UWV. Kansen voor laagopgeleiden. Amsterdam: UWV; 2017.

[41] Morgan DL (1996). Focus Groups. Annu Rev Sociol.

[42] Braun V, Clarke V (2006). Using thematic analysis in psychology. Qual Res Psychol.

[43] Burnard P, Gill P, Stewart K, Treasure E, Chadwick B (2008). Analysing and presenting qualitative data. Br Dent J.

[44] Miles MB, Huberman AM (1994). Qualitative data analysis: an expanded sourcebook.

[45] Ajzen I (1991). The theory of planned behavior. Organ Behav Hum Decis Process.

[46] Sheeran P (2002). Intention—Behavior Relations: A Conceptual and Empirical Review. Eur Rev Soc Psychol.

[47] Huang X. Migrant Workers' Willingness to Participate in Workplace Health Promotion Programs: The Role of Interpersonal and Political Trust in China. Front Public Health. 2020;8(306). 10.3389/fpubh.2020.00306 10.3389/fpubh.2020.00306 PMC7381149 32766198

[48] Robroek SJ, van Lenthe FJ, van Empelen P, Burdorf A (2009). Determinants of participation in worksite health promotion programmes: a systematic review. Int J Behav Nutr Phys Act.

[49] Waterworth P, Pescud M, Chappell S, Davies C, Roche D, Shilton T (2016). Culture, management and finances as key aspects for healthy workplace initiatives. Health Promot Int.

[50] Lier LM, Breuer C, Dallmeyer S (2019). Organizational-level determinants of participation in workplace health promotion programs: a cross-company study. BMC Public Health.

[51] Griffiths F, Lindenmeyer A, Powell J, Lowe P, Thorogood M (2006). Why are health care interventions delivered over the internet? A systematic review of the published literature. J Med Internet Res.

[52] Cook DA, Levinson AJ, Garside S, Dupras DM, Erwin PJ, Montori VM. Instructional Design Variations in Internet-Based Learning for Health Professions Education: A Systematic Review and Meta-Analysis. Acad Med. 2010;85(5). 10.1097/ACM.0b013e3181d6c319 10.1097/ACM.0b013e3181d6c319 20520049

[53] Lindsay Smith G, Banting L, Eime R, O'Sullivan G, van Uffelen JGZ (2017). The association between social support and physical activity in older adults: a systematic review. Int J Behav Nutr Phys Act.

[54] Bukman A, Teuscher D, Feskens E, van Baak M, Meershoek A, Renes R (2014). Perceptions on healthy eating, physical activity and lifestyle advice: opportunities for adapting lifestyle interventions to individuals with low socioeconomic status. BMC Public Health.

[55] Rongen A, Robroek SJ, van Ginkel W, Lindeboom D, Pet M, Burdorf A (2014). How needs and preferences of employees influence participation in health promotion programs: a six-month follow-up study. BMC Public Health.

[56] Boeijinga A, Hoeken H, Sanders J (2016). Health promotion in the trucking setting: Understanding Dutch truck drivers' road to healthy lifestyle changes. Work.

[57] Demou E, MacLean A, Cheripelli LJ, Hunt K, Gray CM (2018). Group-based healthy lifestyle workplace interventions for shift workers: a systematic review. Scand J Work Environ Health.

[58] Velema E, Vyth EL, Steenhuis IH (2017). Using nudging and social marketing techniques to create healthy worksite cafeterias in the Netherlands: intervention development and study design. BMC Public Health.

[59] van Amelsvoort L, de Brouwer CPM, Heerkens YF, Widdershoven GAM, Kant I (2017). Fostering functioning of workers: A new challenge for prevention in occupational health. Work.

[60] Painter SL, Ahmed R, Kushner RF, Hill JO, Lindquist R, Brunning S (2018). Expert Coaching in Weight Loss: Retrospective Analysis. J Med Internet Res.

